# Mercury toxicity risk and corticosterone levels across the breeding range of the Yellow-breasted Chat

**DOI:** 10.1007/s10646-021-02510-6

**Published:** 2022-01-01

**Authors:** Kristen Mancuso, Karen E. Hodges, Manuel Grosselet, John E. Elliott, John D. Alexander, Michelle Zanuttig, Christine A. Bishop

**Affiliations:** 1 Department of Biology, University of British Columbia Okanagan, Kelowna, BC Canada; 2 Tierra de Aves, Veracruz, Mexico; 3 Science and Technology Branch, Environment and Climate Change Canada, Delta, BC Canada; 4 Klamath Bird Observatory, Ashland, OR USA

**Keywords:** Yellow-breasted Chat, Mercury, Corticosterone, Songbird

## Abstract

Mercury (Hg) is an environmental contaminant that can negatively impact human and wildlife health. For songbirds, Hg risk may be elevated near riparian habitats due to the transfer of methylmercury (MeHg) from aquatic to terrestrial food webs. We measured Hg levels in tail feathers sampled across the breeding range of the Yellow-breasted Chat (*Icteria virens*), a riparian songbird species of conservation concern. We assessed the risk of Hg toxicity based on published benchmarks. Simultaneously, we measured corticosterone, a hormone implicated in the stress response system, released via the hypothalamus-pituitary-adrenal axis. To better understand range-wide trends in Hg and corticosterone, we examined whether age, sex, subspecies, or range position were important predictors. Lastly, we examined whether Hg and corticosterone were correlated. Hg levels in chats were relatively low: 0.30 ± 0.02 µg/g dry weight. 148 out of 150 (98.6%) had Hg levels considered background, and 2 (1.6%) had levels considered low toxicity risk. Hg levels were similar between sexes and subspecies. Younger chats (<1 year) had higher Hg levels than older chats (>1 year). Hg levels were lowest in the northern and central portion of the eastern subspecies’ range. Corticosterone concentrations in feathers averaged 3.68 ± 0.23 pg/mm. Corticosterone levels were similar between ages and sexes. Western chats had higher levels of corticosterone than eastern chats. Hg and corticosterone were not correlated, suggesting these low Hg burdens did not affect the activity of the hypothalamus-pituitary-adrenal axis. Altogether, the chat has low Hg toxicity risk across its breeding range, despite living in riparian habitats.

## Introduction

Mercury (Hg) is an environmental contaminant of concern to wildlife and humans because it can negatively affect reproduction, neurology, immunology, and behaviour (Wolfe et al. 1998; Scheuhammer et al. 2007; Seewagen 2010; Fuchsman et al. 2017). Hg from anthropogenic sources (e.g. chlor-alkali plants, coal burning, waste incineration, gold-mining) and naturally occurring sources (e.g. volcanic activity, forest fires) can travel vast distances in the atmosphere before settling in soil and waterways (Driscoll et al. 2007, 2013; AMAP/UN Environment 2019). There, under anoxic conditions, inorganic Hg is transformed by mostly methylating bacteria into its most biohazardous form, methylmercury (MeHg), and readily moves through food webs (Driscoll et al. 2007, 2013; Cristol et al. 2008).

In birds, ingested Hg enters the bloodstream and becomes stored in tissues and organs, primarily the liver, kidneys, brain, and muscles (Braune and Gaskin 1987; Whitney and Cristol 2017a). During the period of feather growth, Hg in blood becomes encapsulated into the feathers and remains inert thereafter (Furness et al. 1986). Hg content in the blood includes a combination of recent dietary exposure and body burden accumulated since the last moult cycle (Honda et al. 1986; Braune and Gaskin 1987; Thompson et al. 1991; Monteiro and Furness 2001; Bottini et al. 2021). Feathers are the main pathway for birds to rid their body of Hg; 70–93% of the body burden gets incorporated into feathers (Honda et al. 1986; Braune and Gaskin 1987; Agusa et al. 2005; Whitney and Cristol 2017a; Albert et al. 2019). Females also depurate Hg through egg-laying (Scheuhammer 1987; Rimmer et al. 2005; Robinson et al. 2012). Therefore, mercury encapsulated in feathers represents the accumuation of Hg between moulting cycles and can provide insight into Hg exposure across the full-annual cycle (Albert et al. 2019).

Recent evidence suggests that songbirds, especially those that use riparian, aquatic, and wetland habitats, may be at risk of Hg exposure through the consumption of carnivorous and aquatic invertebrates (Rimmer et al. 2005; Brasso and Cristol 2008; Edmonds et al. 2010; Evers et al. 2012; Jackson et al. 2015; Ackerman et al. 2016; Pacyna et al. 2017 but see Brasso et al. 2020). Because Hg biomagnifies, songbirds that consume arthropods at higher trophic levels, including spiders, can accumulate elevated levels of Hg (Rimmer et al. 2005; Cristol et al. 2008; Keller et al. 2014; Li et al. 2021).

The identification of songbird populations at risk of Hg toxicity is important because multiple sublethal effects may affect the persistence of a population. Hg reduces body condition and negatively affects the immune function in birds, therefore, individuals may be less equipped to fend off diseases and parasites (Whitney and Cristol 2017b; Ackerman et al. 2019). Hg exposure can negatively affect reproduction through altered nest-building (Chin et al. 2017), reduced courtship behaviour (Heddle et al. 2020), smaller egg size, smaller clutches (Brasso and Cristol 2008, but see Heddle et al. 2020), and lowered nest success through hatchling mortality (Scheuhammer 1987; Custer et al. 2007; Jackson et al. 2011; Heddle et al. 2020). Early developmental exposure to Hg can affect survival, reproduction, behaviour, and neuroanatomical development (Yu et al. 2017; Paris et al. 2018; Heddle et al. 2020). Moreover, Hg may negatively affect migration and overwinter survival, further contributing to the decline of migratory songbirds (Ma et al. 2018; Seewagen 2020).

Of additional concern, Hg can act as a stressor disrupting the normal functioning of the stress response system (Wada et al. 2009; Franceschini et al. 2009; Herring et al. 2012; Moore et al. 2014 but see Tartu et al. 2015a, b; Maddux et al. 2015). The stress response involves the release of glucocorticoids into the bloodstream via adrenal glands through the hypothalamus-pituitary axis (Siegel 1980; Wingfield 2013). In birds, the main glucocorticoid is corticosterone (Siegel 1980; Romero 2004). A wide range of negative effects, including weakened immune systems, hypertension, neuronal cell death, memory loss, and negative fitness consequences are attributed to chronic stress, which can present itself as increased or decreased levels of corticosterone (Busch and Hayward 2009; Kleist et al. 2018).

Like Hg, corticosterone circulating in the blood is deposited into feathers during the period of feather growth and remains inert thereafter (Jenni-Eiermann et al. 2015). In contrast to Hg, corticosterone is a short-lived hormone and feather concentrations represent integrated levels in the blood during the period of growth over the span of several weeks (Bortolotti et al. 2008; Lattin et al. 2011). Measuring Hg and corticosterone in feathers is a non-invasive technique that can be used to address concerns about conservation physiology (Jenni-Eiermann et al. 2015). This is especially advantageous for species or populations that may be at risk and where more invasive methods might not be advised (Bortolotti et al. 2008).

Here, we examine trends in Hg and corticosterone across the breeding range of the Yellow-breasted Chat (*Icteria virens*, hereafter chat). The geographic scope of our study combining Hg and corticosterone in chats is unprecedented. The chat is a neotropical migrant songbird whose habitat consists of dense, shrubby thickets in or near riparian habitats – a habitat zone generally believed to be a hotspot for Hg (Jackson et al. 2015; Eckerle and Thompson 2020). Additionally, given that Hg can transcend aquatic-terrestrial interfaces via invertebrate vectors, the Yellow-breasted Chat may be exposed to Hg through the consumption of invertebrates in these riparian habitats. The diet of the Yellow-breasted Chat consists of terrestrial invertebrates (ants, wasps, spiders, beetles, leafhoppers) and also plant matter (Yard et al. 2004; McKibbin and Bishop 2008; Eckerle and Thompson 2020). Probing deeper into the conservation physiology for this species may prove valuable for conservation efforts, as several populations are of conservation concern in Canada and the United States (British Columbia, Ontario, Environment and Climate Change Canada 2016, 2019; California, Shuford and Gardali 2008; Connecticut, State of Connecticut 2015; New York, New York State 2019). In our study, we measured Hg and corticosterone concentrations in feathers across the breeding range of the chat. Secondly, we converted feather Hg levels to equivalent blood Hg concentrations to assess Hg toxicity risk based on published benchmarks. Thirdly, we explored whether range-wide patterns of Hg and corticosterone could be explained by age, sex, range position, or subspecies. Lastly, we examined the relationship between Hg and corticosterone to see if there was evidence of Hg accumlated since the last moult cycle affecting the function of the hypothalamus-pituitary-adrenal axis.

## Methods

### Study areas

**Fig. 1:**
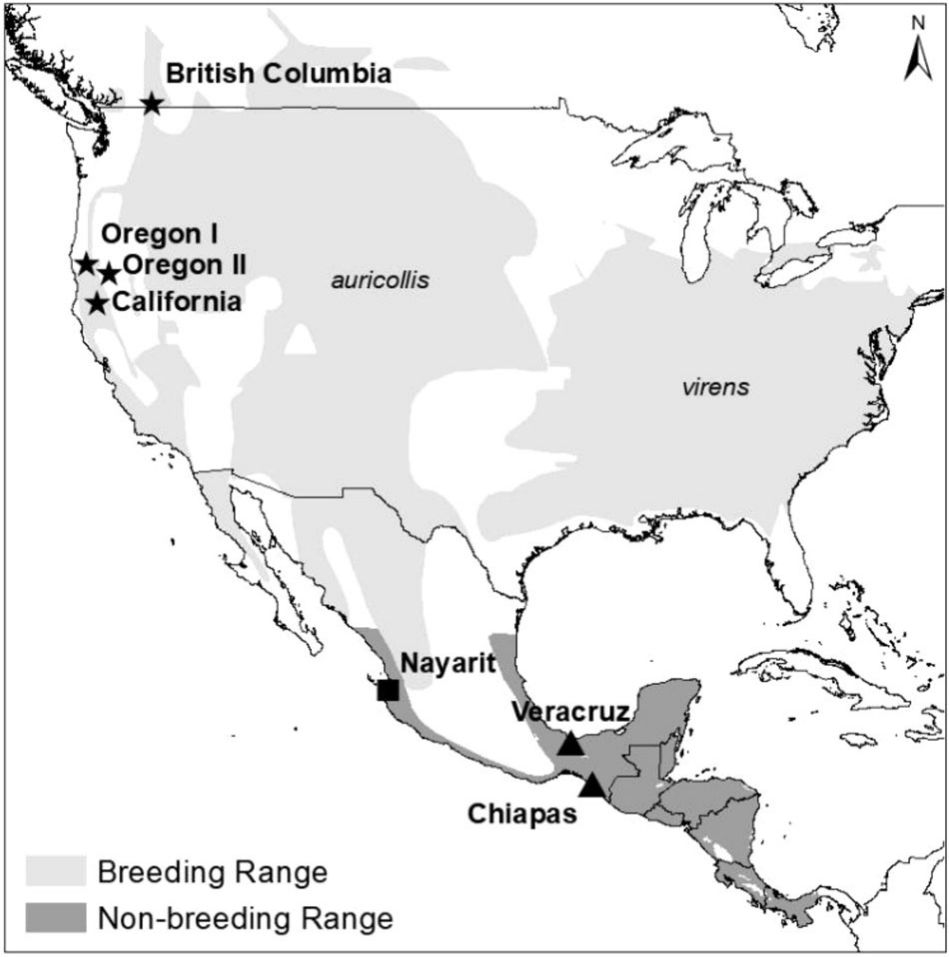
Study areas for Yellow-breasted Chats (*Icteria virens*). The breeding ranges of the eastern (*I.v. virens*) and western (*I.v. auricollis*) subspecies are shown. Star symbols represent birds sampled from study areas in the breeding season, the square represents birds sampled from a study area in the overwintering season, and triangles represent birds sampled from study areas during migration. Map made in ArcMap 10.7.1 (ESRI 2019) using a WGS 1984 Web Mercator projection and coordinate system

We sampled chats in seven study areas. Four areas were known breeding locations in British Columbia, Canada, and California and Oregon, USA (Table 1, Fig. 1). The remaining three study areas were in Mexico in Nayarit, Chiapas, and Veracruz, where chats were sampled during migration and the non-breeding period. Breeding location was inferred from Mexico sites using hydrogen stable isotope analyses and genetics (Mancuso 2020).

**Table 1:** Yellow-breasted Chat (*Icteria virens*) study site locations where feather samples were collected for corticosterone (Cort), mercury (Hg), and hydrogen stable isotope analyses (δ^2^H_f_)

Site	Latitude	Longitude	Elevation (m)	Years	N (individuals sampled)		
					Cort	Hg	δ^2^H_f_
Breeding							
South Okanagan Valley, British Columbia, CA	49.094	−119.537	300	2017, 2018	20	20	15^a^
Trinity River, California, US	40.695	−122.854	500	2017, 2018	16	20	15^a^
WIIM, Oregon I, US	42.490	−123.480	251	2018	8	13	7^a^
TOPS, Oregon II, US	42.019	−122.140	905	2018	3	5	2^a^
Migration							
Santa Alejandrina Bird Observatory, Veracruz, MX	18.002	−94.588	4	2014, 2015	100	36	36
La Encrucijada Biosphere Reserve, Chiapas, MX	15.552	−93.206	3	2018	30	36	36
Overwinter							
San Pancho Bird Observatory, Nayarit, MX	20.905	−105.398	33	2018, 2019	19	20	20
Total					196	150	92

The breeding location of chats sampled during migration and overwintering was inferred using stable hydrogen isotopes

^a^These samples were collected only to calibrate the precipitation isoscape and are not included in the total

All breeding sites were in riparian areas. The British Columbia study area was along the channelized South Okanagan River (McKibbin and Bishop 2010). Three Klamath Bird Monitoring Network (Alexander et al. 2004; Alexander 2011) areas included one along the Trinity River in California (Rockwell and Stephens 2018) and two Oregon study areas, WIIM along the Rogue River and TOPS along the Klamath River (Rockwell et al. 2017). The Nayarit study area consisted of openings and edges of semi-deciduous tropical forests. The Chiapas study site was located within the La Encrujijada Biosphere Reserve near the Pacific Ocean within a large open wetland surrounded by small patches of trees and tall grasses. The Veracruz, Mexico study site was adjacent to a wetland area and surrounded by shrubs and short trees (Gahbauer et al. 2016).

### Bird handling and feather collection

In British Columbia and California, chats were captured and sampled during the breeding season between May and August of 2017 and 2018. In Oregon, chats were captured and sampled in 2018 between May and July but two birds were sampled in September. In Veracruz, chats were sampled in March, May, August to November 2014, and March 2015, and we assumed that these were migrating chats. In Chiapas, chats were sampled in September and October of 2018; we assumed these were migrating chats. In Nayarit, chats were sampled in January and February 2018 and 2019, and we assume these were overwintering chats.

Chats were caught in mist nets often with the aid of call-playback and taxidermied or wooden painted decoys. Birds were removed from nets, sexed according to (Pyle 1997), placed into age categories defined by Wolfe et al. (2010), and banded. Birds that could not clearly be distinguished as male or female were classified as unknown sex. The right outer rectrix feather was collected for corticosterone analyses. The left outer rectrix feather was collected for Hg analyses. One secondary feather (S1–S6) was collected for hydrogen stable isotope analysis. Birds were processed as quickly as possible (a few minutes) and released at the capture site.

### Moult and its interpretation of Hg and corticosterone

Chats have a complex-basic moult strategy. They undergo an incomplete preformative moult during their first annual cycle and a complete definitive prebasic moult during subsequent annual cycles (Pyle 1997; Wolfe et al. 2010). Chats likely complete preformative and definitive prebasic moults on their natal/breeding grounds prior to fall migration (Pyle 1997; pers. obs). The preformative moult in chats includes the outer primaries, inner secondaries, tertials, greater coverts, and up to two inner tail feathers (Pyle 1997). In our western study areas, some chats replace these inner tail feathers during their preformative moult, however, many do not (pers. comm from Matthias Bieber; Pyle 1997). In contrast, Grosselet et al. (2014) found that most eastern chats captured in Veracruz, Mexico had replaced all of their tail feathers during their preformative moults. Therefore, in our study the first cycle outer tail feathers collected for western chats were likely grown in the nest, whereas the first cycle outer tail feathers collected for eastern chats were likely grown on the natal grounds during their preformative moult.

Knowing when and where a chat feather was grown has important implications for our interpretation of Hg and corticosterone. Tail feathers from chats in their first moult cycle encapsulate Hg exposure since hatching (i.e. ~ 10 days for western chats, and up to several months for eastern chats). Feathers from birds in their definitive moult cycle encapsulate Hg exposure over ~1 year since their last moult, including breeding, migration, and the non-breeding season (Evers et al. 2005; Warner et al. 2012; Jackson et al. 2015).

Corticosterone does not accumulate in the body and, therefore, represents the hormonal profile on the breeding grounds during the period of feather growth only (~1–2 weeks), either during the nestling period (e.g. first moult cycle of western chats) or before fall migration (e.g. first moult cycle of eastern chats and all chats in their definitive moult cycle (Bortolotti et al. 2008).

### Breeding assignment of overwintering birds

To determine the breeding origin of chats sampled in Mexico, we measured hydrogen stable isotope ratios in feathers (Mancuso 2020). Hydrogen stable isotope values in precipitation vary latitudinally across the landscape and this chemical signature becomes incorporated into animal tissues (Hobson and Wassenaar 2008). Therefore, by examining the hydrogen stable isotope contents of animal tissue (feathers in this case), one can infer where the tissue was grown (Hobson and Wassenaar 1997).

In brief, feathers were washed in a solvent to remove any impurities, air dried, and cut into small pieces. Feathers were sent to the Laboratory of Stable Isotope Science at the University of Western Ontario, London, Ontario, Canada for analyses. Stable hydrogen isotope composition was determined using an elemental analyzer where samples undergo pyrolysis by heating to 1120 °C. Samples were calibrated using the most recent values for Caribou Hoof Standard and Kudu Horn Standard (Soto et al. 2017). Samples of standard powered keratin (Spectrum-1) were measured intermittently to assess and correct for drift. Random feather samples were run in duplicate for further quality assurance/quality control and were averaged during reporting. The standard notation of stable hydrogen isotopes (δ^2^H) is parts per thousand (‰) relative to Vienna Standard Mean Ocean Water. A subscript F is included when referring to feathers, or P when referring to precipitation hydrogen isotope ratio values.

A precipitation isoscape for North America was created using publicly available δ^2^H_p_ values in rainwater from the Global Network of Isotopes in Precipitation (IAEA/WMO 2019). The package IsoriX (*v0.8.2*; Courtiol et al. 2019) in R (*v3.5.1*.; R Core Team 2018) was used to create the precipitation isoscape and then calibrated using Yellow-breasted Chat feather samples of known breeding origin (Mancuso 2020).

**Fig. 2:**
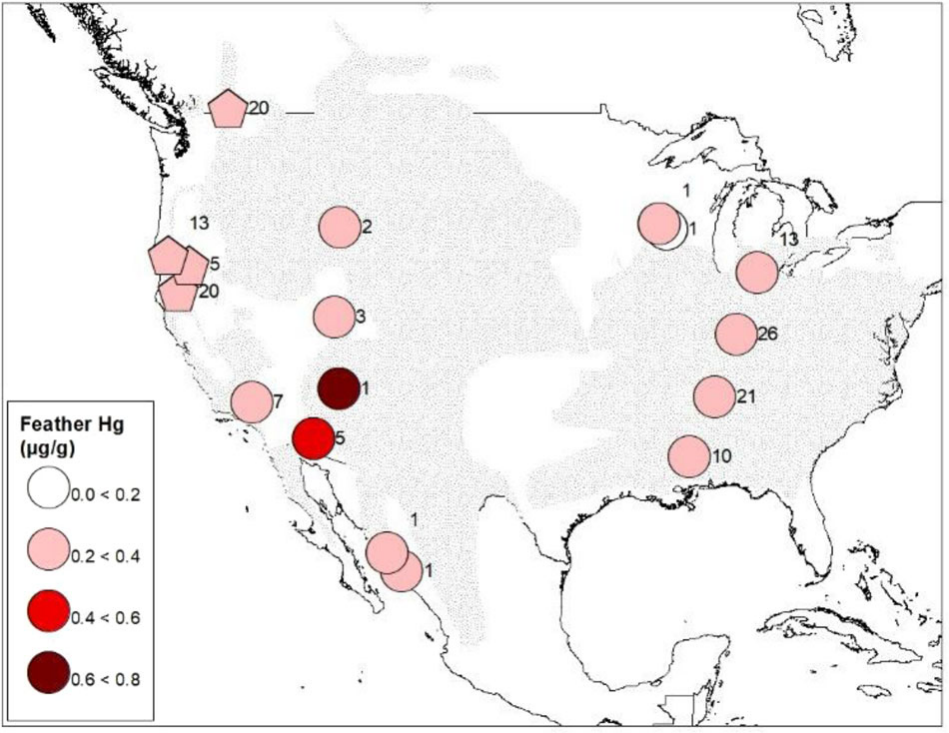
Average mercury concentrations in Yellow-breasted Chats (*Icteria virens*) in their breeding range. Pentagons are sites where chats were sampled during the breeding season. Circles are breeding sites inferred from stable hydrogen isotope analyses; a single point was used to map these inferred groups that span larger areas based on assignment probabilities. Sample sizes are indicated beside each point. The breeding range of the Yellow-breasted Chat is shown in stippled grey in the background (IUCN 2016). Map made in ArcMap 10.7.1 (ESRI 2019) using a WGS 1984 Web Mercator projection and coordinate system. Note that while higher mercury concentrations are shown as darker shades of red, all levels are still considered low risk of mercury toxicity

To estimate the breeding origin of chats, δ^2^H_f_ values were placed into 10‰ bins and a group location was estimated using the isofind function of IsoriX. Group location estimates were exported as a raster file to create maps in ArcMap 10.7.1 (ESRI 2019). Location estimates were constrained to breeding ranges based on subspecies identification previously determined using genetics (Mancuso 2020). The western subspecies (*I.v. auricollis*) breeds in the western United States, southwestern Canada, and northwestern Mexico (Fig. 1, IUCN 2016; Eckerle and Thompson 2020). The eastern subspecies (*I.v. virens*) breeds in the eastern United States and the very southern tip of Ontario, Canada (Fig. 1, IUCN 2016; Eckerle and Thompson 2020). We showed genetically that chats in Chiapas and Veracruz were of the eastern subspecies while chats from Nayarit were of the western subspecies (Mancuso 2020). In order to visualize the geographic assignment locations from the 10‰ hydrogen isotopic bins, a single representative point was used (Appendix I, Fig. 2).

### Hg analyses

We measured total Hg concentration in 150 chat feathers (Table 1). Samples were analyzed by the National Wildlife Research Centre in Ottawa, Ontario, Canada. Feathers were washed with acetone, then a solution of 0.25% Triton X-100, then twice in ultrapure water before being air-dried for ≥48 h. Cleaned samples were freeze-dried and ground into a homogenous powder. Samples were placed into the autosampler on nickel boats, then decomposed under a continuous flow of ultra-pure oxygen within a quartz catalytic tube. The products of combustion were oxidized and carried to a gold amalgamator that targets Hg. Hg is released by heating and carried through absorbance cells within an atomic absorption spectrophotometer. The absorbance was measured at 253.7 nm. Sample values for Hg are reported in µg/g dry weight (dw). As most of the Hg present in feathers is MeHg, total Hg content represents MeHg concentration (Kim et al. 1996; Bond and Diamond 2009; Souza et al. 2020), which is the organic and biohazardous form of Hg.

Accuracy was evaluated at the start of each day with a minimum of four standard reference materials. To maintain accuracy, standard reference materials were analyzed at regular intervals and at the end of each day. The standard reference materials included mussel tissue, lobster hepatopancreas, tuna fish, fish flesh and, human hair (spiked). The average recovery (±SE) of Hg from standard reference materials was 99.3 ± 0.51% across 8 days and 55 tests. The method detection limit was determined using 10 consecutive analyses of a standard reference material containing a low concentration of Hg. The method detection limit as determined on August 12, 2019, based on five mg of SRM Oyster Tissue 1566b was 0.024 ng. Reported method detection limits were adjusted for each sample based on mass and varied between 0.003–0.018 µg/g dw.

### Corticosterone extraction and assay

We followed standard feather corticosterone extraction methods for 197 chat feathers (Bortolotti et al. 2008; Fairhurst et al. 2015; Harris et al. 2016). Each feather was washed with ~1% dilute soap to remove any surface contaminants and then rinsed with ultrapure water. The feathers were air-dried overnight. The calamus was discarded and the length of the feather was measured to the nearest millimetre. The feather was cut into small pieces using fine scissors within a glass test tube and 10 ml of 99.9% HPLC grade methanol was added. Test tubes were covered and sonicated in a water bath at room temperature for 30 minutes and then placed in a heated shaking water bath at 50 °C overnight. The feather pieces were separated from the methanol solution via vacuum filtration into a new glass test tube. A glass funnel plugged with marine PolyWool^TM^ as a filter was fit snug to the filtration funnel using a rubber sleeve. The test tube, feather remnants, and filter were washed twice with 2.5 ml of methanol to capture any remaining corticosterone. The methanol solution was evaporated in a fume hood under air and the extract residues were reconstituted with 300 µl of assay buffer and vortexed 3 times at room temperature. The samples were placed in 1.5 ml centrifuge tubes and centrifuged for ~5 minutes to pelletize any remaining debris or larger molecules. A volume of 100 µl of the supernatant was pipetted into an Enzyme-Linked Immunosorbent Assay plate for each sample.

Corticosterone was assayed using Enzo Life Sciences Enzyme-Linked Immunosorbent Assay kit no. ADI-900-097. This is a competitive binding immunoassay kit specific to the hormone corticosterone that uses a polyclonal sheep corticosterone antibody and a 96 well plate coated with donkey anti-sheep immunoglobulin G. The reported cross-reactivity of these kits include 28.6% deoxycorticosterone, 1.7% progesterone, 0.13% testosterone, 0.28% tetrahydrocorticosterone, 0.18% aldosterone, 0.046% cortisol, <0.03% pregnenolone, <0.03% β-estradiol, <0.03% cortisone, and <0.03% 11-dehydrocorticosterone acetate (Enzo Life Sciences Inc. 2018). The reported sensitivity of the kit is 26.99 pg/mL based on two standard deviations from the blank reading using 16 replicates (Enzo Life Sciences Inc. 2018). We followed the kit instructions to determine corticosterone concentrations, culminating in reading the optical density using a microplate reader set to 405 nm (Enzo Life Sciences Inc. 2018). Samples were run in duplicate and averaged during reporting. Three samples of approximately 50% binding with a concentration of 600 pg/mL were added randomly to each plate to assess inter-assay variability. The inter-assay coefficient of variation was calculated as the standard deviation of the plate means divided by the mean of the means for the 8 plates. The intra-assay coefficient of variation was calculated as the overall average of the standard deviation divided by the mean for each duplicate sample for all samples for 8 plates.

To determine the corticosterone concentration of samples in the plate, the average optical density for the blank cells was subtracted from the optical density for all samples. A 4-parameter logistic model was used to create a standard curve with known corticosterone concentrations of 20,000, 4000, 800, 132, and 32 pg/mL. The drc package (*v. 3.2.1*; Ritz et al. 2015) in programme R was used to create the standard curve, and the unknowns were interpolated along the standard curve using the ED function (Online Resource 1). The concentration of corticosterone within each feather was standardized to feather length and reported in values of pg/mm.

### Hg risk

To examine the risk of Hg toxicity to chats, we compared our Hg values to known blood toxicity benchmarks provided by Ackerman et al. (2016) based on multiple bird species: <0.2 µg/g wet weight (ww) background levels; 0.2–1.0 µg/g ww low risk; 1.0 – 3.0 µg/g ww moderate risk; 3.0 – 4.0 µg/g higher risk; >4.0 µg/g ww severe risk. However, as Ackerman et al. (2016) rankings are based on blood Hg levels, we transformed our dw feather Hg concentrations to equivalent ww blood Hg concentrations.

We used two separate transfer equations from different studies to provide a more comprehensive analysis of our data. Therefore, we had two sets of blood ww Hg equivalents in which to assess against published Hg toxicity benchmarks. The first transfer equation was from Eagles-Smith et al. (2008), who reviewed Hg levels across many tissue types for 4 waterbird species in San Francisco Bay, California. This equation was appealing because it was based on a large number of individuals and several species, however, the species were not landbirds. We used the transfer function reported for breast feathers because this equation was used to adjust between feathers and blood in Ackerman et al. (2016). The equation was: ln(blood Hg) = ln(feather Hg)*0.673 – 1.673.

The second transfer function was from Jackson et al. (2011) for Carolina Wrens (*Thyrothorus ludovicianus*). This equation was appealing because the study was on an insectivorous landbird and outer tail feathers were used, however, it was based on a single species and in a contaminated site (Jackson et al. 2011). The re-arranged equation for blood was: blood Hg = (tail feather Hg - 0.64)/3.38. Using the results from these two transfer functions separately, we calculated the percent of total samples that fell within each risk category.

### Statistical analysis

To determine which factors influence Hg and corticosterone levels in chats, we used an information-theoretic approach comparing the fit of multiple models using Akaike’s Information Criterion adjusted for small sample sizes (AIC_c_, Burnham and Anderson 2002). All values are reported as mean ± standard error unless otherwise noted. Both Hg and corticosterone were transformed before analyses to improve normality and model fit as both variables were highly positively skewed. Hg was log_e_ transformed. Corticosterone was transformed to the power of −0.7 based on a Tukey’s Ladder of Powers transformation to produce a more normal distribution. Transformed variables were assessed with a Shapiro–Wilk’s test to verify normality.

For Hg samples (*n* = 150), we tested 12 models containing different combinations of the parameters: age, sex, subspecies, and range location (Table 2). Age included two categories: first cycle and definitive cycle. Two chats in unknown plumage were lumped into the definite cycle age group. Age relates to the accumulation of Hg since the previous moult. First cycle western chats presumably represent natal Hg exposure as a nestling (~1–2 weeks). First cycle eastern chats presumably represent Hg exposure on the natal grounds after leaving the nest (~weeks to months). Definitive cycle chats represent accumulation over the full annual cycle, including breeding, migration, and overwintering (~1 year).

**Table 2:** Models compared to predict mercury in Yellow-breasted Chat (*Icteria virens*) feathers

Model	K	Log-Likelihood	AICc	Δ AIC_c_	Weight
Age + Range position	8	−137.00	291.03	0.000	0.754
Age + Sex + Range Position	10	−136.45	294.49	3.458	0.134
Age + Subspecies	4	−144.14	296.55	5.518	0.048
Subspecies × Age	5	−143.60	297.61	6.584	0.028
Age	3	−145.91	297.98	6.953	0.023
Age + Sex + Subspecies	6	−143.91	300.40	9.372	0.007
Sex + Age	5	−145.69	301.80	10.773	0.003
Intercept-only model	2	−150.27	304.62	13.587	0.001
Range position	7	−145.11	305.00	13.970	0.001
Subspecies	3	−149.57	305.31	14.284	0.001
Sex	4	−149.95	308.18	17.154	0.000
Sex + Range position	9	−144.47	308.23	17.199	0.000
Sex + Subspecies	5	−149.24	308.90	17.874	0.000

K is the number of parameters. AIC_c_ is Akaike’s Information Criterion adjusted small sample sites. The total sample size is 150. The response variable, mercury (μg/g) in Yellow-breasted Chat feathers, was log_e_ transformed to improve model fit

Sex included male, female, and unknown and was included to assess potential differences in diet and the effects of Hg depuration through egg-laying (Scheuhammer 1987; Robinson et al. 2012; Tartu et al. 2015a).

Subspecies was used as a way to compare exposure levels between eastern and western North America as well as examine any differences between subspecies. Note that the eastern subspecies occurs generally in part of the American midwest, the Great Lakes region and southward towards Texas, while the western subspecies includes central and the western United States and western Mexico (Fig. 1).

Range location was included to examine if differences in Hg exposure occurred in different geographic areas on a smaller scale than subspecies. The range classes were chosen to reflect the broad geographic assignments from isotopes while maintaining adequate sample sizes for each category for statistical analyses. Range location included six categories based on subspecies and general position within the breeding range. The western north range included samples from British Columbia, and δ^2^H_f_ values ≤ −80‰ for the western subspecies (Appendix 1). The western central range location included samples from California, Oregon, and δ^2^H_f_ values from −50 > −80‰ for the western subspecies. The western southern range location included δ^2^H_f_ values −10 > −50‰ for the western subspecies. The eastern north range included δ^2^H_f_ values ≤ −50‰ for the eastern subspecies. The eastern central range included δ^2^H_f_ values −40 > −50‰ for the eastern subspecies. The eastern southern range included δ^2^H_f_ values −20 > −40‰ for the eastern subspecies.

An interaction between subspecies and age was included to account for the potential difference in preformative moult between subspecies as described earlier. All factors were fixed factors because we were specifically interested in examining the effects of each factor level of each predictor on Hg levels in feathers.

For corticosterone samples (*n* = 196), models were conducted in the same way as for Hg. Minor differences for the age category for corticosterone samples was that the first cycle category contained one western chat in juvenal plumage and one western chat moulting into its formative plumage. Two chats in unknown plumage were lumped into the definitive cycle age category. Age, sex, subspecies, range location, and the interaction between age and subspecies were included as model parameters. We tested twelve exploratory models with different combinations of these predictors (Table 3).

**Table 3:** Comparing models to explain corticosterone in Yellow-breasted Chat (*Icteria virens*) feathers

Model	K	Log-Likelihood	AIC_c_	Δ AIC_c_	Weight
Subspecies	3	115.69	−225.26	0.000	0.455
Age + Subspecies	4	116.95	−223.58	1.672	0.197
Age × Subspecies	5	115.84	−223.48	1.782	0.187
Sex + Subspecies	5	116.02	−221.72	3.539	0.078
Age + Sex + Subspecies	6	116.19	−219.94	5.320	0.032
Range position	7	117.26	−219.92	5.335	0.032
Age + Range position	8	117.41	−218.05	7.208	0.012
Sex + Range position	9	117.61	−216.26	8.995	0.005
Age + Sex + Range position	10	117.77	−214.34	10.91	0.002
Intercept-only model	2	104.48	−204.9	20.36	0.000
Age	3	105.10	−204.08	21.18	0.000
Sex	4	105.49	−202.77	22.49	0.000
Sex + Age	5	105.85	−201.38	23.88	0.000

K is the number of parameters. AIC_c_ is Akaike’s Information Criterion adjusted small sample sites. The total sample size is 196. Note that the response variable is corticosterone (pg/mm) in Yellow-breasted Chat feathers was transformed to the power of −0.7 to improve model fit

Linear models were created using the lme4 package (*v.1.1.21*; Bates et al. 2015) in R. We inspected plots of the model residuals versus fitted values for the full model and for the top models to verify that the assumptions of linear models were met. The fit of each model was compared using the MuMIn package (*v1.43.15*; Barton 2019). We report ΔAIC values and relative weights for each model (Burnham and Anderson 2002). Conditional plots of the top model were implemented using the visreg package (v2.6.0; Breheny and Burchett 2017). If the top model had a weight of less than 0.90, suggesting model uncertainty, then parameter estimates were calculated using a model averaging approach (Burnham and Anderson 2002). We computed the average model parameter estimates and 95% confidence intervals by factoring in the relative weight of the models using the model.avg function in MuMln. Parameter estimates shown are from the subset method, where only those models containing the parameters are used in estimation (Barton 2019). We also estimated parameters using the full method, where all models are used in estimation, and the results were similar and, therefore, are not reported. While our statistical approach used transformed values of corticosterone and Hg as indicated to meet model assumptions, we also present original values for visualization.

To examine whether there was a relationship between Hg and corticosterone (*n* = 127 birds where both were sampled), we ran a correlation analysis using the mosaic package (*v.1.5.0;* Pruim et al. 2017) in R for all birds, plus separately for birds in different age classes. To meet the assumption of bivariate normality, Hg values were log_e_ transformed and corticosterone values were inversely transformed before analyses. Shapiro–Wilk’s tests were conducted on the transformed variables to confirm normality.

## Results

Stable hydrogen isotope values spanned between −17.85‰ and −90.38‰ (Appendix 1). The most depleted bins represent samples originating from the northern part of the chat’s breeding range, and the least depleted bins represent samples originating from the southern part of their breeding range (Appendix 1).

### Hg

The average Hg levels in feathers across all chats were 0.30 ± 0.02 µg/g dw. All samples were greater than the method limit of detection.The conversion of our feather Hg values to equivalent blood values using the equation from Jackson et al. 2011 resulted in 139 out of 150 (92.7%) samples with negative values which were nonsensical. All values from Eagles-Smith et al. (2008) were above 0. Regardless, the two transfer functions converting feather Hg to the equivalent blood levels resulted in the same number of chats falling within each Hg risk category. Almost all (98.6%) chats had blood equivalent Hg levels considered background (<0.2 μg/g ww blood equivalent). Only two chats (1.3%) were in the low Hg toxicity risk category of 0.2–1 μg/g (ww blood equivalent), one of which originated from the Oregon I study area, and the other in the southwestern region (δ^2^H_f_: −30 < −40‰, Appendix 1). No samples fell within the moderate, high, or severe risk categories for blood equivalent Hg toxicity.

Our isotopic assignments from overwintering sites combined with known breeding sites provided wide coverage of the chat breeding range (Fig. 2, Appendix 1). However, geographic assignments from stable hydrogen isotopes are broad geographic areas and therefore, we cannot say with high precision where exact breeding occurred or Hg exposure. The two breeding sites with chats having the highest average Hg concentrations occurred in the southwest (Fig. 2). The sample size varied widely between breeding sites, with several sites represented by only 1 sample and so average values representing an entire area should be interpreted with caution.

**Fig. 3:**
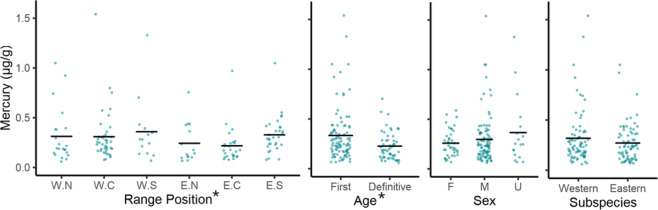
Mercury concentrations in Yellow-breasted Chat (*Icteria virens*) feathers. Untransformed data and means are shown. The right outer tail feather was used. Range position is divided by subspecies (Western, W and Eastern E) and position within range (North, N; Central, C; and Southern, S). Sample sizes are W.N = 22, W.C = 42, W.S = 14, E.N = 15, E.C = 26, E.S = 31. Age includes the first moult cycle (*n* = 97) and the definitive moult cycle (*n* = 53). Sexes are female (F, *n* = 38), male (M, *n* = 94), and unknown (U, *n* = 18). The total sample size is 150. Parameters identified the top model are denoted with asterisks

Hg levels in chat feathers were best explained by a top model that included age and range position as predictors (Table 4, Online Resource 2). This model explained little variance in Hg levels in chats as the adjusted R^2^ value for this model was 0.13. The weight of the top model was 75.4% suggesting that there is some uncertainty about model selection, therefore, all models were factored in for parameter estimation. Hg levels were lowest for chats in their definitive moult cycle (parameter estimate: −0.44 ± 12 relative to first cycle moult; 95% CI −0.67 to −0.21, Table 4, Fig. 3). Hg levels were lowest in the eastern subspecies’ northern (parameter estimate: −0.43 ± 0.22; 95% CI −0.86 - −0.01) and central range locations (parameter estimate: −0.46 ± 0.19; 95% CI −0.82 to −0.09, Table 4, Fig. 3). No statistical differences were found between sexes or subspecies as these parameter estimates included 0 (Table 4, Fig. 3).

**Table 4:** Weighted parameter estimates for mercury concentrations in Yellow-breasted Chats (*Icteria virens*) based on all models using the subset method

	Estimate	Std. error	CI lower	CI upper
**(Intercept)**^**a**^	−**1.21**	**0.16**	−**1.52**	−**0.91**
**Age—definitive cycle**	−**0.44**	**0.12**	−**0.67**	−**0.21**
Range position—West Central	−0.03	0.17	−0.35	0.30
Range position—West South	0.11	0.21	−0.30	0.53
**Range position—East North**	−**0.43**	**0.22**	−**0.86**	−**0.01**
**Range position—East Central**	−**0.46**	**0.19**	−**0.82**	−**0.09**
Range position—East South	0.11	0.18	−0.24	0.45
Sex—male	0.10	0.13	−0.14	0.35
Sex—unknown	0.16	0.18	−0.20	0.51
Subspecies—Eastern	−0.20	0.12	−0.41	0.01
Subspecies Eastern × Age—definitive	0.23	0.23	−0.22	0.68

The 95% confidence intervals that do not include 0 are in bold

^a^The default values for categorical parameters are: Age = first cycle, Subspecies = western, Sex = female, Range position = Western – North. All parameter estimates are relative to the intercept

### Corticosterone

Corticosterone concentrations in feathers were on average 3.68 ± 0.23 pg/mm. One sample was slightly outside the range of the standard curve (i.e. >20,000 pg/ml maximum) and therefore, the corticosterone concentration could not be calculated with confidence. Field notes show this bird had an unusual dark grey skin tone and may have had an underlying health issue; we omitted this sample, and our final sample size was 196. The inter-assay coefficient of variation was 11.3% and the intra-assay coefficient of variation was 3.2%. No samples fell below the level of sensitivity.

**Fig. 4:**
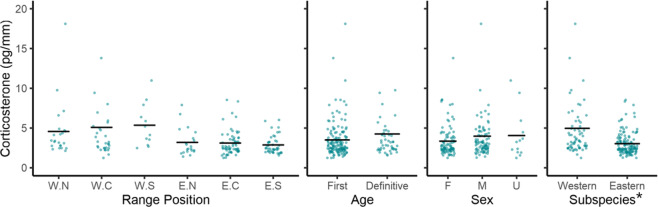
Corticosterone concentrations in Yellow-breasted Chat (*Icteria virens*) feathers. Untransformed data and means are shown. The right outer tail feather was used. Range position is divided by subspecies (Western, W and Eastern E) and position within range (North, N; Central, C; and Southern, S). Sample sizes are W.N = 23, W.C. = 31, W.S. = 12, E.N. = 21, E.C = 64, E.S. = 45. Age includes the first moult cycle (*n* = 148) and the definitive moult cycle (*n* = 48). The total sample size is 196. Note that one outlier with corticosterone concentration of 35.53 pg/mm is not visible. The parameter identified in the top model is denoted with an asterisk

The top model for corticosterone included subspecies as a predictor with a weight of 46.8% (Table 3, Online Resource 2). The adjusted R^2^ value of 0.091 indicates this model explained very little of the variance in feather corticosterone. Two other models were within 2 ΔAIC_c_ units of the top model and included age + subspecies, and the interaction between age and subspecies as predictors and had model weights of 19.8% and 18.7%, respectively (Table 3). Regardless, the low weight of the top model indicates there is some degree of model uncertainty, and therefore, we factored in all models for parameter estimates. Corticosterone levels were lowest for the eastern subspecies (parameter estimate: −0.09 ± 0.02 relative to the wastern subspecies; 95% CI: −0.14 - −0.05) and accordingly, all range positions for the eastern subspecies (Table 5, Fig. 4). Despite age being a parameter in the 2^nd^ and 3^rd^ top models, differences in corticosterone levels between the different age classes of chats were not statistically meaningful as the 95% confidence interval estimates included 0. No differences were detected in corticosterone levels between sexes (Fig. 4).

**Table 5:** Weighted parameter estimates for corticosterone concentrations in Yellow-breasted Chats (*Icteria virens*) based on all models using the subset method

	Estimate	Std. error	CI lower	CI upper
**(Intercept)**^**a**^	−**0.41**	**0.02**	−**0.45**	−**0.37**
**Subspecies—Eastern**	−**0.09**	**0.02**	−**0.14**	−**0.05**
Age—definitive	0.00	0.03	−0.06	0.07
Subspecies—Eastern × Age—Definitive	−0.07	0.05	−0.16	0.02
Sex—male	0.01	0.02	−0.04	0.05
Sex—unknown	−0.03	0.04	−0.11	0.05
Range position—West Central	−0.02	0.04	−0.10	0.05
Range position - West South	0.05	0.05	−0.04	0.15
**Range position—East North**	−**0.09**	**0.04**	−**0.18**	−**0.02**
**Range position—East Central**	−**0.09**	**0.03**	−**0.17**	−**0.03**
**Range position—East South**	−**0.11**	**0.04**	−**0.18**	−**0.04**

The 95% confidence intervals that do not include 0 are in bold

^a^The default values for categorical parameters are: Age = first cycle, Subspecies = western, Sex = female, Range = West North. All parameter estimates are reported relative to the intercept

### Hg and corticosterone combined

**Fig. 5:**
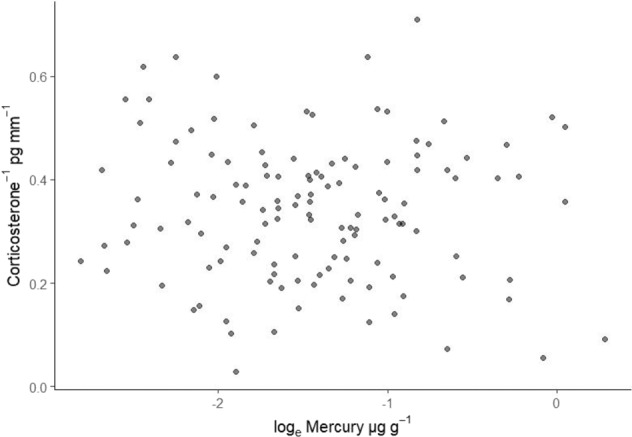
Correlation between mercury and corticosterone in Yellow-breasted Chat (*Icteria virens*) feathers. Feathers represent breeding sites throughout North America (*N* = 127). Both variables were transformed to meet assumptions of normality. Pearson r = −0.054, df = 125, *p* = 0.54

We had 127 feather samples where both Hg and corticosterone were measured on the same individual. There was no correlation between Hg and corticosterone in chat feather samples (Fig. 5, r = −0.054, df = 125, 95% CI: −0.223, 0.121, P-value = 0.54). When the correlation analysis was repeated separately for birds in different age classes, still no correlation was detected (first cycle birds: r = −0.103, df = 82, 95% CI: −0.310, 0.114, P-value = 0.35; definitive cycle birds: r = −0.037, df = 41, 95% CI: −0.333, 0.267, *P*-value = 0.82).

## Discussion

We examined Hg concentrations in chats across their breeding range and found no evidence that this species is at risk of Hg toxicity despite their affinity for riparian habitats. Across all sites, Hg concentrations in chat feathers were within the range of equivalent blood Hg levels considered to be background, except for 2 out of 150 which were low risk of Hg toxicity (Ackerman et al. 2016). Age and range position were predictors for Hg in chats on a range-wide scale. Hg levels were greater in younger birds in their first moult cycle compared to older birds in their definitive moult cycle. Hg levels were lowest in eastern chats that had bred in the north and central portion of their range. Subspecies was an important predictor for corticosterone in chats on range-wide scale. Corticosterone levels were greater in eastern chats than western chats. Hg and corticosterone levels were not correlated, suggesting no evidence of Hg affecting the normal function of the hypothalamus-pituitary-adrenal axis.

### Hg toxicity risk

Hg levels in chat feathers were relatively low, 0.30 ± 0.02 μg/g dw. The average level of Hg in tail feathers for chats in our study is slightly lower than the 0.4 – 0.7 μg/g dw averages reported for other songbirds in terrestrial habitats without a known point source of contamination (Óvári et al. 2018; Ma et al. 2018; Stenhouse et al. 2020). The two chats that fell within the low-risk category for blood equivalent levels (0.2–1.0 μg/g ww) were at the low end of this range with equivalent blood Hg levels of less than 0.3 μg/g ww. These two chats are at minimal risk because most published effects within this category occur at blood Hg levels of 0.3 μg/g ww or greater (Ackerman et al. 2016). The only indication of a harmful effect of Hg at that level was reported in Lesser Scaup (*Aythya affinis*) experiencing some degree of oxidative stress at blood levels equivalent to 0.2 μg/g ww (Custer et al. 2000; Ackerman et al. 2016). Likewise, all values we converted to blood equivents were lower than the reproductive effect thresholds in for small birds of 2.1–4.2 μg/g ww summarized from a meta-analysis by Fuchsman et al. (2017), further suggesting that chats are at little to no risk of mercury toxicity effects. Additionally, Jackson et al. (2015) had two blood samples from chats in contaminated sites in the Appalachian forests with a mean of 0.099 μg/g ww suggesting that even in contaminated sites, Hg toxicity risk is low for chats.

We found no evidence of chats being at risk of Hg toxicity, despite living in riparian habitats, where Hg exposure can be greater as a result of feeding on aquatic insects from contaminated sediments or through aquatic-terrestrial food web chains involving predatory invertebrates (Brasso and Cristol 2008; Cristol et al. 2008). The association with riparian or wetland habitats alone does not necessarily always indicate high Hg risk (Brasso et al. 2020). Brasso et al. (2020) advised caution on the generalization that songbirds in wetland habitats are at greater risk of Hg bioaccumulation after conducting a literature review. While chats consume both invertebrates and plants, it is likely that most invertebrates were of low trophic status or not linked to aquatic food webs. One study found that less than 2% of the chat diet was aquatic insects (Yard et al. 2004).

### Range-wide Hg predictors

Females had slightly lower levels of Hg than males, although not statistically so, and this likely is a function of adult females depurating a relatively small amount of Hg (compared to feathers) into eggs (Honda et al. 1986; Rumbold et al. 2001; Agusa et al. 2005). Dietary intake of Hg is likely similar between sexes and this is not unexpected as we have not observed any sex-based differences in foraging habits and no studies on diet have indicated any sex-based differences (McKibbin and Bishop 2008; Eckerle and Thompson 2020). Other studies on songbirds have also found no difference in Hg levels between sexes (Warner et al. 2012; Keller et al. 2014).

It was unexpected that younger chats (in their first moult cycle) had higher levels of Hg than older chats (in their definitive moult cycle). Because Hg accumulates in the body between moulting periods (Evers 2018; Albert et al. 2019), we expected older birds to have the highest levels of Hg but our results do not support this idea. This trend has been reported elsewhere for other songbirds (Keller et al. 2014; Ma et al. 2018). One purported explanation for this phenomenon is that nestling birds have high energetic demands, especially for protein-rich foods, and, therefore, may be consuming higher volumes of insects and spiders, compared to older birds who may be eating proportionally more plant-based foods (Warner et al. 2012; Ma et al. 2018). This is likely true for chats because nestling chats are fed predominantly adult and larval insects (Schadd and Ritchison 1998) and occasionally berries (McKibbin and Bishop 2008) while adult chats consume roughly equal proportions of invertebrates and fruit (Howell 1932; Eckerle and Thompson 2020).

While our geographic assignments from hydrogen stable isotopes preclude precise estimates chat breeding location, the highest levels in our study generally occurred in western chats that had bred in the southwest. Hg hotspots have been identified in this area (Ackerman et al. 2016). Hg levels across the United States between 2013–2017 were highest in the central and western regions (National Atmospheric Deposition Program 2020). Hotspots of Hg exist in Nevada and California due to the gold rush and Hg mining (Commission for Environmental Cooperation 1997; Rytuba 2000; Alpers et al. 2005) and elevated Hg levels have been observed in birds in these regions (Henny et al. 2002; Hothem et al. 2008). Likewise, agricultural wetlands in the Central Valley of California, notably for rice production, use hydrological regimes that further promote MeHg production, more so than in naturally occurring wetlands (Windham-Myers et al. 2014). As Hg levels for adult chats in our study represent levels accumulated across the full annual cycle, western chats were likely exposed to these areas during migration and during breeding for some populations (Mancuso 2020). Chats may have also been exposed to higher levels of Hg during the non-breeding season in Mexico or Central America, as has been documented for the Bicknell’s Thrush (Rimmer et al. 2005) from past and ongoing use of Hg in gold mining in the region (Canham et al. 2021). Additional studies at different parts of the annual cycle (e.g. using blood samples) would further elucidate where exposure is greatest as in our current study we cannot pinpoint where exposure occurred. Levels of Hg in chats were lowest in the eastern chat range, which was unexpected given that point sources of emissions for anthropogenic sources of Hg are greater in the eastern United States compared to the western United States (Eagles-Smith et al. 2016; AMAP/UN Environment 2019; Steenhuisen and Wilson 2019). It is also possible that Hg levels varied due to geographic differences in diet or trophic level (Keller et al. 2014; Ackerman et al. 2019; Li et al. 2021), but this would also require more in-depth study. Regardless, overall Hg levels in chats in our study were of low concern for toxicity, despite small-scale differences in Hg levels in breeding birds in various locations across their range.

We acknowledge that there have been mixed recommendations on using feathers, especially flight feathers, to draw conclusions about Hg exposure in birds (Bond and Diamond 2008; Peterson et al. 2019; Low et al. 2020). Hg levels in feathers generally correspond with the order of moult, with the first-moulted feathers containing the highest Hg levels (Furness et al. 1986; Bottini et al. 2021; Gatt et al. 2021). Most passerines replace their tail feathers about halfway through their moult beginning with the innermost tail feathers (Pyle 1997). Therefore, using tail feathers likely underestimates the true body burden of chat Hg concentrations accumulated since the last moult, but these are common feathers used in analyses and therefore are suitable for comparison (Warner et al. 2012; Pacyna et al. 2017; Óvári et al. 2018). We believe that the strength of our study is that the same methods were employed across all individuals in their range, allowing for suitable relative comparisons of Hg exposure between individuals and populations (Bortolotti 2010).

### Range-wide corticosterone predictors

We found no differences in corticosterone levels in Yellow-breasted Chats of different ages or sexes but we did find differences between the eastern and western subspecies. Causes for these differences require further study, but possibilities include differences in life history and behaviour, which were suspected for differences in corticosterone for subspecies of Swamp Sparrows (*Melospiza georgiana*, Angelier et al. 2011). While statistically we found a difference in corticosterone levels between subspecies, the magnitude of the differences between the eastern and western are slight and therefore are unlikely to be meaningful in a biologically relevant context. Further investigation such as body condition or reproductive success would help to better contextualize potential differences between subspecies in relation to conservation physiology (Rich and Romero 2005; Dickens and Romero 2013; Boves et al. 2016).

The difficulty in predicting corticosterone is demonstrated by the top model having a very low R^2^ value, explaining very little of the variation in corticosterone in chats. Since the chats in our study were free-living wild birds, a multitude of other factors may influence corticosterone concentrations that we could not control, such as inclement weather (Wingfield et al. 1983; Ouyang et al. 2012), the presence of predators (Romero and Wingfield 2015), food abundance (Busch and Hayward 2009), injury (Sapolsky et al. 2000), human disturbance (Strasser and Heath 2013), immune status (Busch and Hayward 2009), and habitat quality (Marra and Holberton 1998), all of which vary in time and space. Quantifying such factors would be virtually impossible over such a large geographic area.

There is controversy on the usefulness of this hormone for conservation physiology, in particular, as an indication of stress in an organism (Romero 2004; Dickens and Romero 2013; Harris et al. 2017; MacDougall-Shackleton et al. 2019). As the main function of glucocorticoids relates to energy mobilization, levels of these hormones are not synonymous with stress (Busch and Hayward 2009; MacDougall-Shackleton et al. 2019). Additionally, levels of corticosterone vary drastically from species to species, and therefore, direct comparison of chats to other songbirds is not meaningful (Romero 2004), and therefore we did not compare our values to other species. Measures of body condition are often useful as a covariate in studies examining stress, as a decrease in mass has been the only consistent finding associated with chronic stress (Rich and Romero 2005; Dickens and Romero 2013). In our study, incorporating body condition was not possible because relevant body condition would have needed to be assessed the year prior during moult when corticosterone was incorporated into the feathers we collected. Regardless, our study provides range-wide baseline data on the functioning of the hypothamalus-pituitary-adrenal axis on the natal and breeding grounds for this species.

### Relationship between Hg and corticosterone

We did not find any evidence of a correlative relationship between Hg and corticosterone in chats. Studies on Turkey Vultures (*Cathartes aura*) and Zebra Finches have also failed to find an increase in corticosterone with increasing Hg levels (Moore et al. 2014; Maddux et al. 2015; Herring et al. 2018). In contrast, a negative correlative relationship between corticosterone and Hg was detected in Forster’s Terns (*Sterna forsteri*, Herring et al. 2012) and Tree Swallows (Franceschini et al. 2009), where high Hg levels correlated with low baseline corticosterone levels. The true association between these chemicals is unclear as results are inconsistent between studies (Whitney and Cristol 2017b) and may also be related to additional environmental contaminants, such as lead (Herring et al. 2018).

We acknowledge that a limitation in our study of using feathers to compare corticosterone and Hg is the different time frames these two signatures represent. Hg in feathers is accumulated since the last moult (up to a year for birds in their definitive moult cycle), while corticosterone is an integrated level over the period of feather growth (~weeks). Despite the mismatch in the timeframe for adult birds, Hg accumulates in the body and is highest at the time of moult (Albert et al. 2019), and as such we would expect the potential negative effects of Hg on the hypothalamus-pituitary-adrenal axis and subsequent release of corticosterone to be most prominent during that time. The utility of correlations for physiological measures has also been disputed (Bortolotti 2010) but may be important starting points for future investigations when significant trends are detected. The geographic scale at which we compare corticosterone and Hg in the same individuals is unprecedented and allows for a broader understanding of these two biochemicals across various populations.

## Conclusions

In conclusion, our study was the first range-wide study to examine both Hg and corticosterone in a free-living terrestrial songbird. While it is generally thought that organisms associated with wetland or riparian habitat are at higher risk of Hg exposure, we found that in the absence of a point source of contamination, chats are at low risk of Hg toxicity across their breeding range. Additionally, we found no evidence of Hg disrupting the normal function of the hypothalamus-pituitary-adrenal axis. Given that several populations of chats are of conservation concern, our results are reassuring that Hg and corticosterone are unlikely to be factors impeding their recovery.

## Supplementary Information

Suppl Mtrls 1

Suppl Mtrls 2

## Data Availability

The datasets supporting the conclusions of this article will be available in the Open Science Framework repository.
