# Supplementary material for: Mercury toxicity risk and corticosterone levels across the breeding range of the Yellow-breasted Chat
Source: Ecotoxicology. 2022 Jan 1;31(2):234–50. doi: 10.1007/s10646-021-02510-6 (PMC8901494; doi:10.1007/s10646-021-02510-6)

Mercury toxicity risk and corticosterone levels across the breeding range of the Yellow-breasted Chat

Ecotoxicology Journal

Kristen Mancuso^1*^, Karen E. Hodges^1^, Manuel Grosselet^2^, John E. Elliott^3^, John D. Alexander^4^, Michelle Zanuttig^3^, Christine A. Bishop^3^.

***Corresponding Author:** [kmancuso88@gmail.com](mailto:kmancuso88@gmail.com). ORCID: 0000-0003-4702-2250

**Supplementary Material 1**

Four parameter logistic curves based on known standards were used to interpolate corticosterone levels in Yellow-breasted Chat feathers. A total of 8 Enzo Life Sciences Enzyme-Linked Immunosorbent assays plates for corticosterone (kit no. ADI-900-097) were used and the corresponding standard curves and parameter estimates are shown for each plate. The black dots in each graph are the known standards, and the blue triangles show the samples interpolated along the curve. The corticosterone concentration of the standards were 32, 160, 800, 4,000, and 20,000 pg/ml., The samples represent the mean corticosterone concentration (based on two samples) in 100 μl of sample. Corticosterone values reported in the manuscript were standardized to feather length and reported in pg/mm.


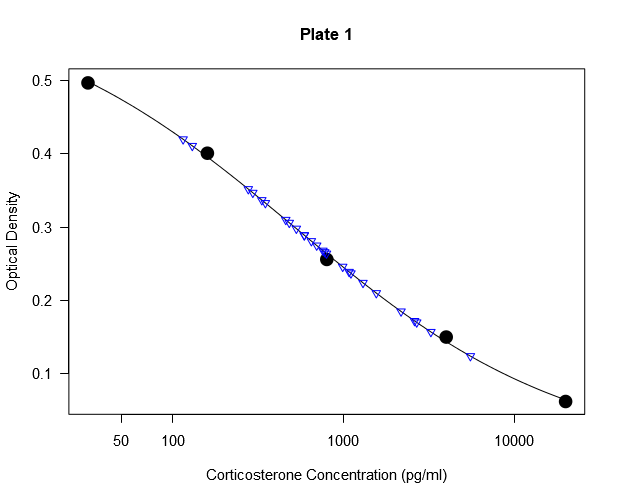


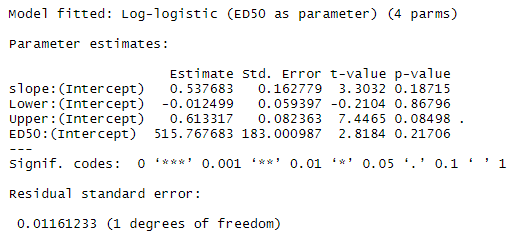


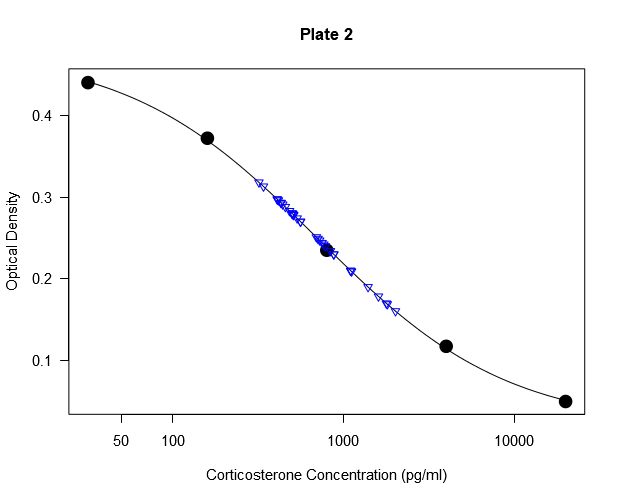


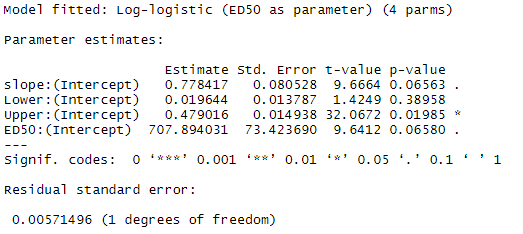


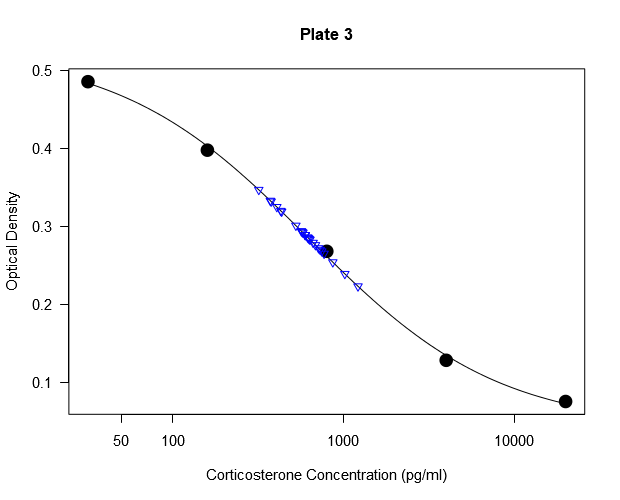


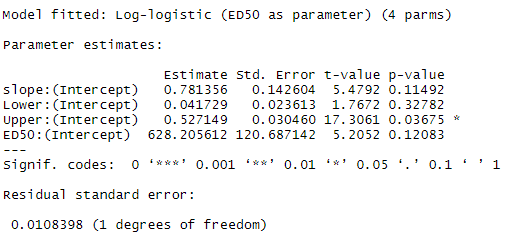


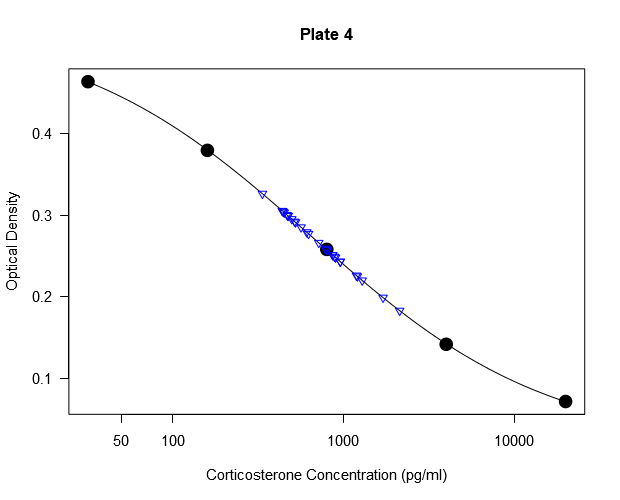


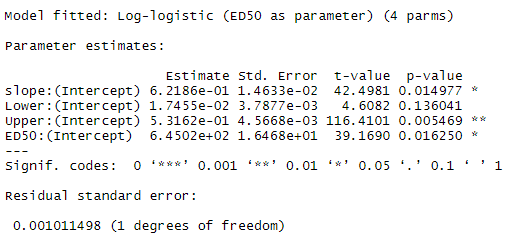


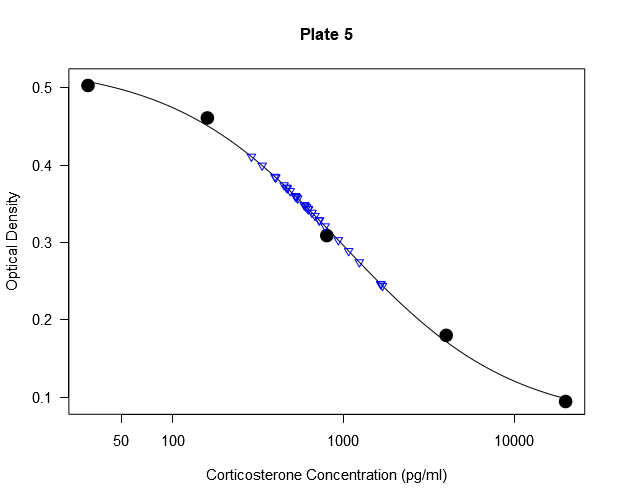


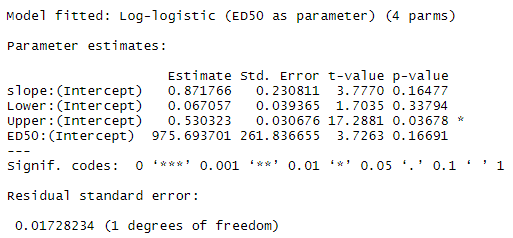


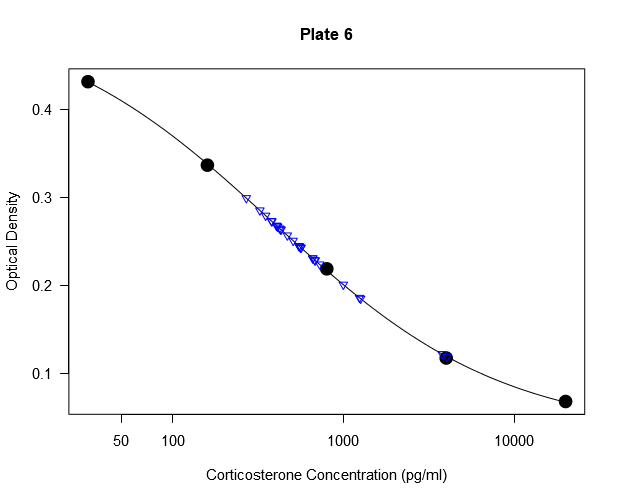


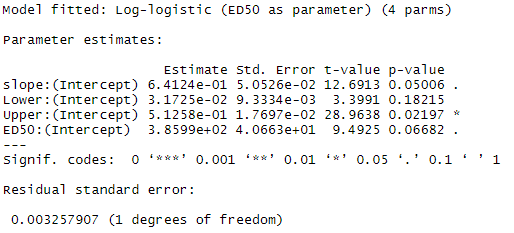


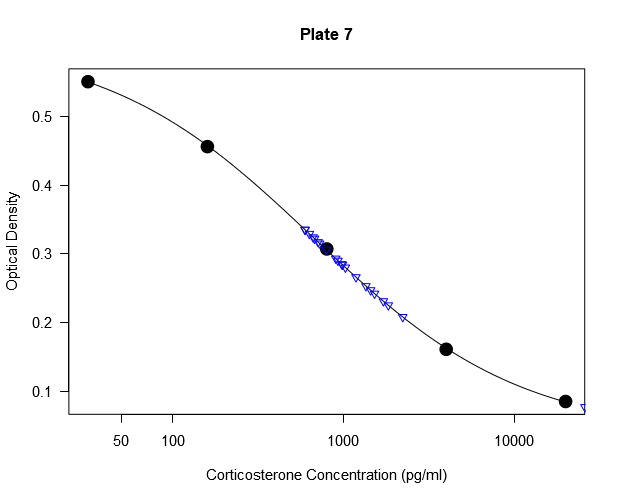


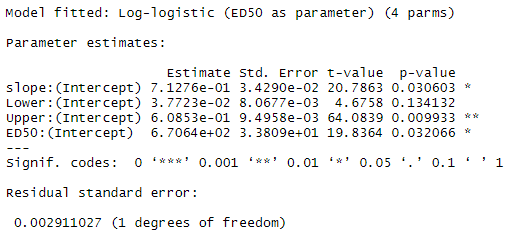


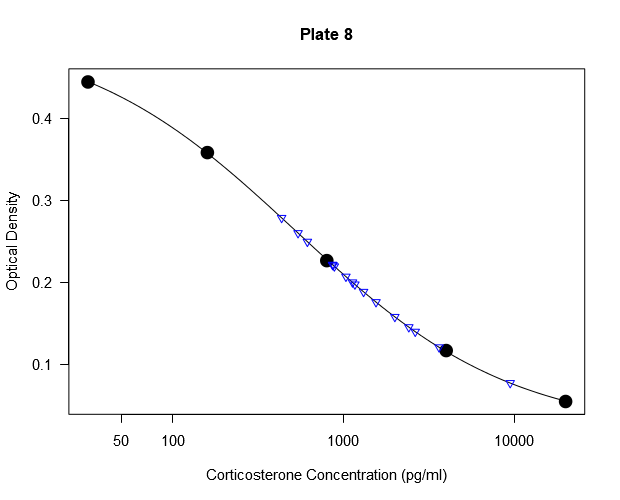


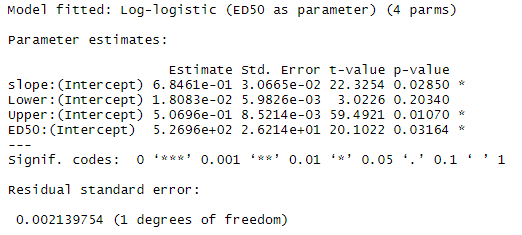

Supplement: Supplementary file 1 — Suppl Mtrls 1 [file 10646_2021_2510_MOESM1_ESM.docx]
