# Supplementary material for: Mercury toxicity risk and corticosterone levels across the breeding range of the Yellow-breasted Chat
Source: Ecotoxicology. 2022 Jan 1;31(2):234–50. doi: 10.1007/s10646-021-02510-6 (PMC8901494; doi:10.1007/s10646-021-02510-6)

Mercury toxicity risk and corticosterone levels across the breeding range of the Yellow-breasted Chat

Ecotoxicology Journal

Kristen Mancuso^1*^, Karen E. Hodges^1^, Manuel Grosselet^2^, John E. Elliott^3^, John D. Alexander^4^, Michelle Zanuttig^3^, Christine A. Bishop^3^.

***Corresponding Author:** [kmancuso88@gmail.com](mailto:kmancuso88@gmail.com). ORCID: 0000-0003-4702-2250

**Supplementary Material 2**

Conditioning plots of the top model explaining feather mercury and corticsterone in Yellow-breasted Chats, separately. The model shown for the first two plots is: Feather Mercury ~ Age + Range Position, where feather mercury has been log_e_ transformed. The upper plot shows the conditioning plot for age while the second plot shows the conditioning plot for range position. The top model shown for the third plot is: Feather Corticosterone ~ Subspecies, where feather corticosterone has been transformed to the power of -0.7. Each plot displays partial residuals (black dots), prediction lines (blue line), and 95% confidence bands (gray band).


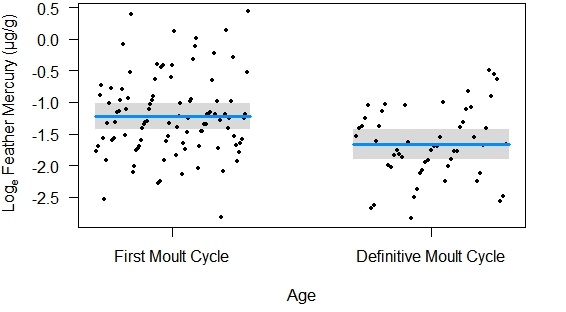

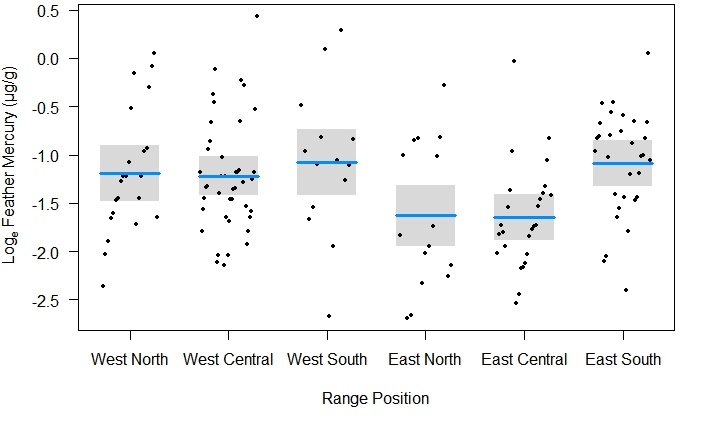


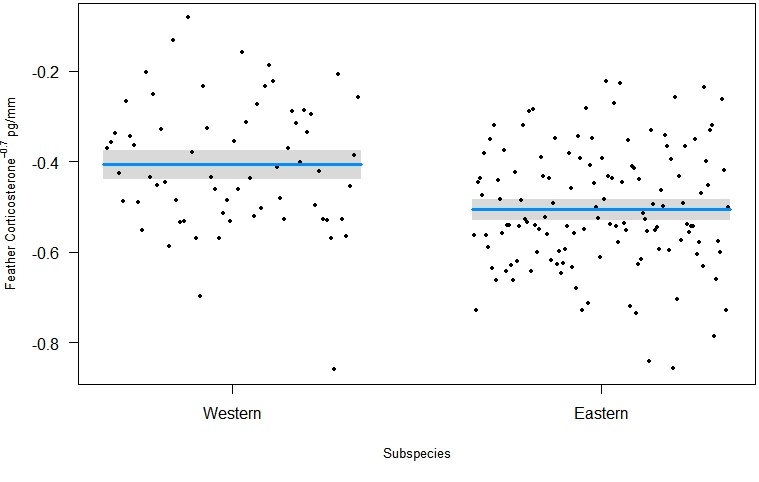

Supplement: Supplementary file 2 — Suppl Mtrls 2 [file 10646_2021_2510_MOESM2_ESM.docx]
